# Effects of Placenta-Derived Mesenchymal Stem Cells on the Particulate Matter-Induced Damages in Human Middle Ear Epithelial Cells

**DOI:** 10.1155/2019/4357684

**Published:** 2019-11-14

**Authors:** So Young Kim, Seung Ha Oh, Jun Ho Lee, Myung-Whan Suh, Moo Kyun Park

**Affiliations:** ^1^Department of Otorhinolaryngology-Head & Neck Surgery, CHA University College of Medicine, Republic of Korea; ^2^Department of Otorhinolaryngology-Head & Neck Surgery, Seoul National University College of Medicine, 101 Daehak-Ro Jongno-Gu, Seoul 110-744, Republic of Korea

## Abstract

This study was aimed at investigating the effects of placenta-derived mesenchymal stem cells (PL-MSCs) on particulate matter- (PM-) exposed human middle ear epithelial cells (HMEECs). HMEECs were treated with 300 *μ*g/ml PM for 24 hours. The PL-MSCs were cocultured with PM-treated HMEECs. Cells were harvested on days 0, 1, and 4, and the expression of the inflammatory genes *TNFα*, *COX2*, *IL1β*, *IL6*, and *MUC5B* in HMEECs and anti-inflammatory genes *PTGES*, *TGFβ*, and *VEGF* in PL-MSCs was examined by qRT-PCR. The culture media were collected to measure the secreted PGE2 level using an enzyme-linked immunosorbent assay. The mRNA expression of *TNFα*, *COX2*, *IL1β*, *IL6*, and *MUC5B* in HMEECs increased following PM treatment. PM-treated HMEECs cocultured with PL-MSCs showed alleviated inflammatory reactions represented by lower mRNA expression levels of *MUC5B*, *TNFα*, *IL1β*, and *IL6* compared to monocultured PM-treated HMEECs. The mRNA expression levels of *PGE2*, *TGFβ*, and *VEGF* were elevated in cocultured PL-MSCs compared to those of control PL-MSCs. The medium of PM-treated HMEECs cocultured with PL-MSCs exhibited increased PGE2 levels. The increased inflammatory response in PM-treated HMEECs was reversed using PL-MSCs. The *PGE2*, *TGFβ*, and *VEGF* were the mediators of the anti-inflammatory effects of PL-MSCs.

## 1. Introduction

Particulate matter (PM) is a major airway pollutant that has increased in recent years. Enormous adverse health outcomes have been documented for airway, cardiovascular, neurological, and metabolic disorders [1, 2]. It has been suggested that PM elevates inflammation and oxidative stress and impairs cell proliferation [3, 4]. The middle ear is connected to the upper airway via the Eustachian tube. Thus, PM could enter directly into middle ear epithelial cells and cause injury. In addition, the blood supply of the middle ear epithelium could be a route for PM-induced effects. Prior studies have demonstrated inflammation in human middle ear epithelial cells (HMEECs) following PM exposure [5, 6]. The 24 hours of diesel exhaust particle (DEP) increased the mucin-producing gene of MUC5AC in HMEECs [7]. However, few studies have explored the temporal changes in PM effects on the middle ear. In addition, the anti-inflammatory response of stem cells following PM exposure in the middle ear has not been well-described.

Several natural compounds are believed to relieve PM-induced inflammation [8]. However, the rescue effects from PM-induced inflammation are questionable. Moreover, the active compounds and their mechanisms of action have not been delineated. Mesenchymal stem cells (MSCs) secrete several trophic factors with immunomodulatory, antiapoptotic, and anti-inflammatory effects [9–11]. For instance, an *in vitro* study reported that the adipose-derived mesenchymal stem cells (AD-MSCs) exerted anti-inflammatory effects on osteoarthritic chondrocytes and synoviocytes after 3 days of coculture [9]. Several animal studies have revealed the therapeutic roles of MSC secretomes in the immune system and inflammation [12–14]. Because the middle ear is a readily accessible route for drug delivery, topical administration of MSCs could readily control middle ear pathologies.

The hypothesis of the present study was that placenta-derived MSCs (PL-MSCs) could restore PM-induced middle ear epithelial inflammation via trophic factors released from activated PL-MSCs without direct contact with injured cells and that genes from injured cells may activate PL-MSCs. To verify this, PM-induced HMEEC inflammation was examined with the related genes. Next, PM-exposed HMEECs were cocultured with PL-MSCs without direct contact. The preliminary results were presented at the International Congress of ORL-HNS 2017 (ICORL 2017) [15]. In addition, the expression of anti-inflammatory and proliferative genes was measured. The inflammatory changes in MSCs after coculture with PM-injured HMEECs were also examined.

## 2. Materials and Methods

### 2.1. Cell Culture

**Figure 1:**
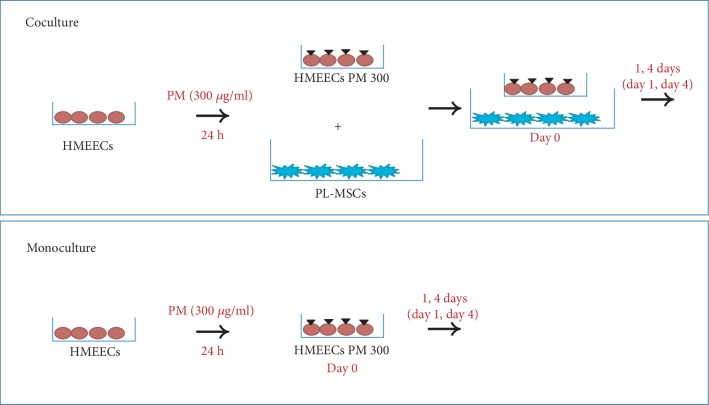
Experimental design of the present study. Human middle ear epithelial cells (HMEECs) were incubated with particulate matter (PM) for 24 hours. HMEECs were then cocultured with placenta-derived mesenchymal stem cells (PL-MSCs) for 1 and 4 days.

HMEECs were obtained from Dr. David J. Lim at the House Ear Institute (Los Angeles, CA, USA). HMEECs were cultured in a mixture of Dulbecco's Modified Eagle's Media (DMEM; Invitrogen, Carlsbad, CA, USA) and bronchial epithelial basal media (1 : 1; Lonza, Walkersville, MD, USA). Cells were maintained at subconfluence in a CO_2_-enriched (95% air, 5% CO_2_) humidified atmosphere at 37°C. PL-MSCs were obtained from CHA Biotech Co. Ltd. (South Korea). PL-MSCs were cultured in alpha-MEM (Gibco, Gaithersburg, MD, USA) containing 10% fetal bovine serum (Gibco), 100 U/ml penicillin, and 100 *μ*g/ml streptomycin in a CO_2_-enriched (95% air, 5% CO_2_) humidified atmosphere at 37°C. PM (Standard Reference Material (SRM) 2975; National Institute of Standards and Technology, Gaithersburg, MD, USA) was suspended in sterile saline (0.9% NaCl) containing Triton X (0.001%). Prior to the experiments, PM was sonicated for 15 min and vortexed to minimize aggregation. HMEECs were treated with 200 or 300 *μ*g/ml PM for 24 hours. Next, coculture was performed by incubating HMEECs in transwells (0.4 *μ*m pore size; Corning® Transwell® polyester membrane cell culture inserts (Cat. No. COP-3450)) and PL-MSCs in the lower chamber of a 6-well plate at a 1 : 1 cell ratio (Figure 1). We set day 0 for the initial day of coculture. Before 300 *μ*g/ml PM-treated HMEECs were cocultured with PL-MSCs with transwells, HMEECs were washed three times using PBS. HMEECs and PL-MSCs were cocultured in the media used for HMEEC culture. Control cells were monocultures of PL-MSCs and HMEECs. To analyze the direct effects of PM on PGE2 secretion of PL-MSCs, the PL-MSCs were monocultured with treatment of 300 *μ*g/ml PM. Coculture of PL-MSCs and HMEECs was repeated four times. On days 0, 1, and 4, both monocultures and cocultures were detached with trypsin-EDTA, and viability was determined using the trypan blue exclusion method and the MTT assay to measure the cytotoxic effects of PM in HMEECs. Cells were harvested on days 0, 1, and 4 for quantitative reverse transcription polymerase chain reaction (qRT-PCR) analysis of *mucin 5B* (*MUC5B*), *tumor necrosis factor alpha* (*TNFα*), *interleukin 1 beta* (*IL1β*), *interleukin 6* (*IL6*), *cyclooxygenase 2* (*COX2*), *prostaglandin E synthase* (*PTGES*), *transforming growth factor beta* (*TGFβ*), and *vascular endothelial growth factor* (*VEGF*), and supernatants were stored at -80°C for enzyme-linked immunosorbent assay (ELISA) of prostaglandin E2 (PGE2).

### 2.2. Analysis of Gene Expression

Total RNA was extracted from human PL-MSC and HMEEC monocultures and cocultures using TRI Reagent® (Sigma-Aldrich, St. Louis, MO, USA) according to the manufacturer's instructions. Reverse transcription was performed using TOPscript™ RT DryMix (dT 18 plus; Enzynomics Co. Ltd., Daejeon, South Korea) according to the manufacturer's protocol. Forward and reverse oligonucleotides for PCR amplification of *MUC5B*, *TNFα*, *IL1β*, *IL6*, *COX2*, *PTGES*, *TGFβ*, and *VEGF* are shown in Table 1. RT-PCR was run in a Bio-Rad-CFX96 Touch™ Real-Time PCR Detection System using TOPreal™ qPCR 2x PreMIX (SYBR Green with low ROX; Enzynomics) with the following protocol: initial activation of HotStarTaq DNA polymerase at 95°C for 15 min, followed by 45 cycles of 95°C for 10 s and 60°C for 15 s. The amplification efficiency (E) of each amplicon was determined using 10-fold serial dilutions of positive control complementary DNAs (cDNAs) and calculated from the slopes of the log input amounts (from 20 ng to 2 pg of cDNA) plotted versus the crossing point values according to the formula *E* = 10^–1/slope^. All primer efficiencies were confirmed to be high (>90%) and comparable (Table 1). For each target gene, messenger RNA levels were calculated, normalized to *GAPDH* according to the formula 2^–Ct^, and are expressed as a percentage of the reference gene. All RT-PCRs were replicated three times for each gene for four respective coculture sets.

Cell culture media were collected following 4 days of cultures and monocultures, and the secretion of prostaglandin E2 (PGE2) was measured using an ELISA (Prostaglandin E2 Parameter Assay Kit, R&D Systems, Minneapolis, MN, USA) according to the manufacturer's instructions.

### 2.3. Statistical Analysis

Statistical analysis was performed using the Mann–Whitney *U* test. SPSS v.21.0 software (IBM, Armonk, NY, USA) was used for analysis. *P* values less than 0.05 were considered significant.

## 3. Results

### 3.1. Effects of PM on HMEECs

**Figure 2:**
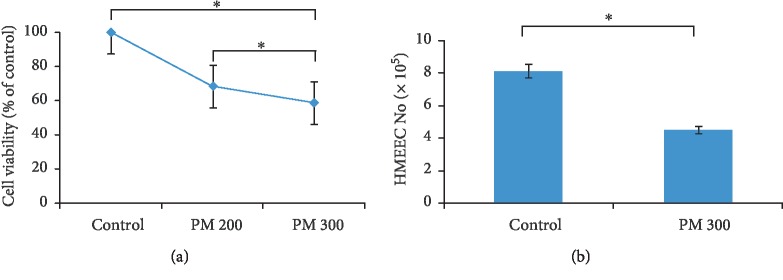
The changes of viable human middle ear epithelial cells (HMEECs) after the particulate matter (PM) treatment. (a) The number of viable HMEECs decreased after the PM treatment in the MTT assay. (b) The changes of cell viability in trypan blue counting (^∗^*P* < 0.05 of the Mann–Whitney *U* test for monoculture conditions, ^∗∗^*P* < 0.05 of the Mann–Whitney *U* test between monoculture and coculture conditions).

**Figure 3:**
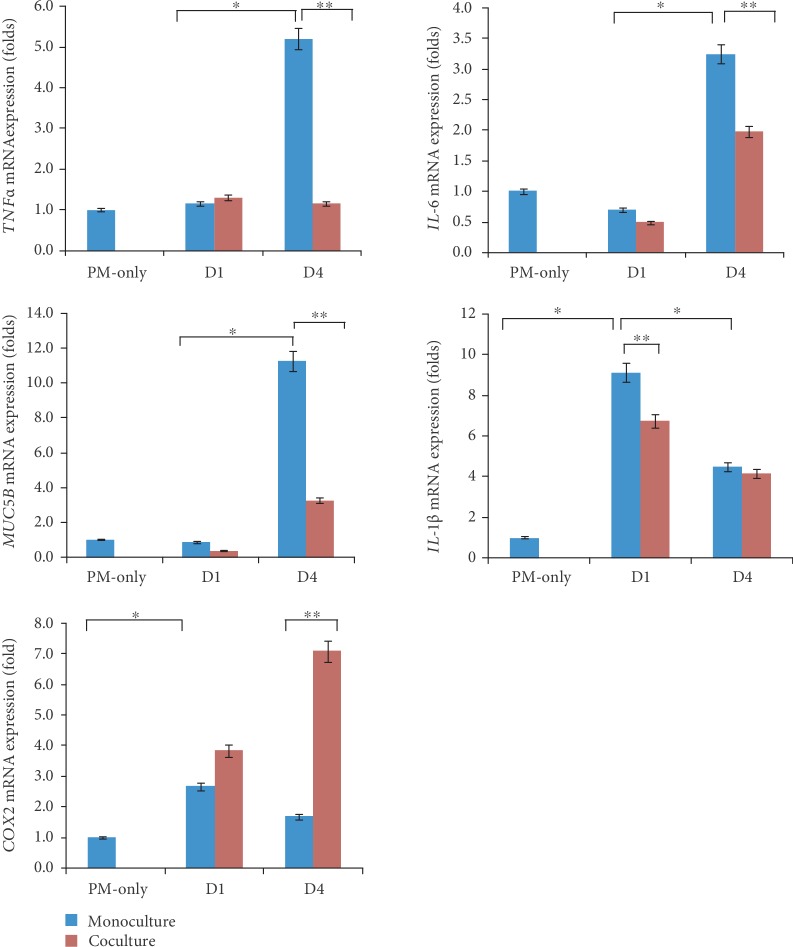
Changes in mRNA expression levels of human middle ear epithelial cells (HMEECs) after particulate matter (PM) treatment and coculture with placenta-derived mesenchymal stem cells (PL-MSCs). The mRNA levels of *IL1β*, *TNFα*, *IL6*, *COX2*, and *MUC5B* were increased after PM treatment. The increased expression of *IL1β*, *TNFα*, *IL6*, and *MUC5B* was alleviated after coculture with PL-MSCs. The expression of *COX2* was higher under coculture conditions than monoculture conditions (*interleukin 1 beta* (*IL1β*)*, tumor necrosis factor alpha* (*TNFα*), *interleukin 6* (*IL6*), *cyclooxygenase 2* (*COX2*), and *mucin 5B* (*MUC5B*)).

The cell viability was significantly decreased after 200 or 300 *μ*g/ml of 24-hour PM exposures, compared to control HMEECs (Figure 2(a)) (*P* = 0.03 for 200 *μ*g/ml PM and *P* = 0.02 for 300 *μ*g/ml PM). Trypan blue staining also showed a significant decrease of viable HMEECs in the PM-exposed groups (Figure 2(b)). The mRNA expression of *TNFα*, *IL6*, *MUC5B*, *IL1β*, and *COX2* was differently increased in HMEECs following incubation with 300 *μ*g/ml PM at different time points (Figure 3). The mRNA expression of *TNFα*, *IL6*, and *MUC5B* was significantly increased at day 4 (5 days after PM treatment). The *IL1β* and *COX2* expression peaked on day 1 (2 days after PM treatment) and decreased on day 4.

### 3.2. Effects of PL-MSCs on PM-Treated HMEECs

**Figure 4:**
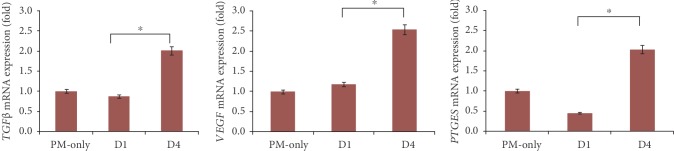
Changes in mRNA expression levels of placenta-derived mesenchymal stem cells (PL-MSCs) after coculture with particulate matter- (PM-) treated human middle ear epithelial cells (HMEECs). The mRNA levels of *TGFβ*, *VEGF*, and *PTGES* were increased after coculture with PM-treated HMEECs (*transforming growth factor beta* (*TGFβ*), *vascular endothelial growth factor* (*VEGF*), and *prostaglandin E synthase* (*PTGES*)).

**Figure 5:**
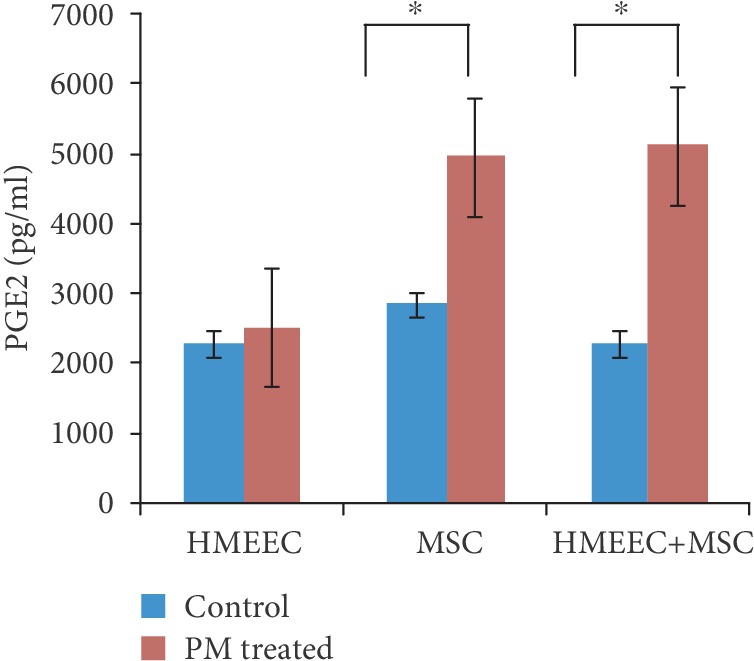
Secreted PGE2 levels in culture media. The PGE2 enzyme-linked immunosorbent assay demonstrated elevated secretion of PGE2 in PM-treated placenta-derived mesenchymal stem cell (PL-MSC) coculture media (prostaglandin E2 (PGE2)).

Coculture with PL-MSCs caused a significant decrease in the mRNA expression of *TNFα*, *IL1β*, *IL6*, and *MUC5B* in HMEECs compared to monocultured HMEECs at 4 days of culture with PL-MSCs (Figure 3). In contrast, *COX2* mRNA expression was more elevated under coculture conditions than monocultured HMEECs. The mRNA expression of *TNFα* and *COX2* was not significantly changed in PL-MSCs after coculture with PM-treated HMEECs for 0, 1, and 4 days (Supplementary [Supplementary-material supplementary-material-1]). *TGFβ*, *PTGES*, and *VEGF* mRNA expression was increased in PL-MSC after coculture with PM-treated HMEECs for 44 days (Figure 4). *TGFβ* and *VEGF* mRNA expression was not significantly changed in PM-treated HMEECs after coculture with PL-MSCs for 0, 1, and 4 days (Supplementary [Supplementary-material supplementary-material-1]). On the other hand, *PTGES* mRNA expression in PM-treated HMEECs increased 1 day after coculture with PL-MSCs. This increase decreased after 4 days of coculture with PL-MSCs in PM-treated HMEECs. In the culture medium, secreted PGE2 was not increased in monocultured PM-treated HMEECs, while it was significantly elevated under coculture conditions (Figure 5).

## 4. Discussion

### 4.1. Principle Findings

PM exposure decreased the proliferation of HMEECs. The inflammatory genes *TNFα*, *IL1β*, *IL6*, and *COX2* and the mucin-producing genes of *MUC5B* were elevated in HMEECs following treatment with PM. After coculture with PL-MSCs, the mRNA expression of *TNFα*, *IL1β*, and *IL6* was decreased in HMEECs and that of *TGFβ*, *PGE2*, and *VEGF* was increased in PL-MSCs. Few studies have reported the rescue effects of PL-MSCs in PM-induced epithelial injuries. In addition, the present study observed temporal changes in gene expression profiles in both HMEECs and PL-MSCs. The administration of MSCs into the middle ear could be a potential therapeutic tool for middle ear inflammation or otitis media based on this study. The optimal duration of administered MSCs needed to exert anti-inflammatory effects is expected to be 2-4 days from this study. There are no therapies for otitis media except for antibiotics for bacterial infection. Thus, the MSCs could be used to rescue the inflamed and injured middle ear epithelium in the otitis media of patients. Although the optimal MSC treatment conditions need to be explored in future studies, the local delivery of drug via the middle ear may be promising, as described in previous studies [16, 17].

### 4.2. Expression of Inflammatory Genes in PM-Treated HMEECs

PM initiated the inflammatory response in HMEECs in the present study. The mRNA expression of *IL1β* and *COX2* was elevated 2 days after exposure to PM (day 1). Leukotrienes and prostaglandins are some of the earliest types of cytokines produced in response to antigenic stimulation. COX2 produces prostaglandin and elevates its expression in the otitis media [18]. After 5 days of PM exposure (day 4), levels of the inflammatory genes *TNFα* and *IL6* were increased. *TNFα* is an inflammatory gene that induces inflammation in the middle ear and upregulates mucin gene expression and subsequent mucin secretion [19]. Indeed, the mRNA expression of *MUC5B* increased after 5 days of PM exposure (day 4). MUC5B is related to mucociliary clearance [20]. The middle ear epithelium of chronic otitis media patients expresses MUC5B [21]. These temporal changes of inflammatory responses of HMEECs after PM exposure may be in line with increased activation of reactive oxygen species on day 4 compared to day 2 in monocultured HMEECs (Supplementary [Supplementary-material supplementary-material-1]).

### 4.3. Attenuated Inflammatory Response in PM-Treated HMEECs

The inflammatory response following PM exposure was relieved under PL-MSC coculture conditions. Because there was no direct contact between HMEECs and PL-MSCs, the mediating cytokines could interact between cells. The mRNA levels of the inflammatory genes *TNFα* and *IL6* were decreased in HMEECs after coculture with PL-MSCs for 4 days. The mRNA expression of *TGFβ*, *PGE2*, and *VEGF* was increased in PL-MSCs when cocultured with PM-treated HMEECs. Moreover, it was presumed that the PL-MSC effects are dependent on existing inflammation. The levels of anti-inflammatory genes were elevated in PL-MSCs along with the increase of inflammatory genes in PM-treated HMEECs. Small effects were observed in cells that produce low levels of inflammatory genes on day 1 of coculture. However, both the inflammatory and anti-inflammatory genes in HMEECs and PL-MSCs were markedly changed after 5 days. Previous studies reported that human MSCs require activation, and activating stimuli appear to include the proinflammatory genes *IL1β* and *TNFα* [22]. It was also reported that the anti-inflammatory effects of MSCs are independent of the tissue sources or MSC donors and result from the inflammatory status of target cells [9]. In the presence of inflammatory stimuli, PL-MSCs relieve the inflammation by releasing anti-inflammatory genes.

### 4.4. Modulation of the COX2/PGE2 Pathway by PL-MSCs

Although several inflammatory (*TNFα* and *IL6*) and anti-inflammatory (*TGFβ* and *VEGF*) genes altered their expression level in accordance with PM exposure and coculture conditions, the changes in *COX2* and PGE2 were remarkable. The high expression of PGE2 during coculture with PL-MSCs suggests that the COX2/PGE2 pathway acts as a modulator of PL-MSC actions. In contrast to other inflammatory genes, *COX2* mRNA expression was more augmented under PL-MSC coculture conditions, which may have been caused by the increased mRNA expression of *COX2* in response to elevated PGE2 production [23]. Previous studies have also suggested that the therapeutic immunomodulatory effects of MSCs correlate with COX2-derived PGE2 expression [9, 10, 14]. The paracrine secretion of PGE2 from umbilical cord MSCs primarily affects acute lung injuries in mice [14]. PGE2 plays a major role in the immunomodulatory actions of MSCs. Although MSCs constitutively secrete PGE2 without the need for activation signals [10, 24, 25], the present results demonstrated accelerated secretion of PGE2 following the increase in inflammatory genes. These results confirm that exogenous PGE2 is sufficient to inhibit TNF*α* secretion from inflammatory-activated cells [10, 26]. In summary, the increased secretion of PGE2 from cocultured PL-MSCs may decrease *TNFα* expression in PM-exposed HMEECs, thereby alleviating the inflammatory response.

### 4.5. Limitations of the Present Study

The PL-MSC coculture showed orchestrated changes in gene profiles in both HMEECs and PL-MSCs. However, we did not conduct a long-term coculture experiment in this study due to the limitations of the transwell system. From day 4 of the coculture condition, PL-MSCs showed decreased proliferation and morphological changes. *VEGF* mRNA expression was decreased after day 4 with senescence of PL-MSCs under the coculture conditions. PL-MSCs and HMEECs were incubated in transwells with the culture medium of HMEECs to reflect the clinical conditions of the middle ear. Although there was senescence of PL-MSCs, they exhibited anti-inflammatory effects in coculture conditions. Although the increased protein level of PGE2 was demonstrated using ELISA assay, other trophic gene products were not assessed in the present study. These anti-inflammatory effects may also depend on other factors released by PL-MSCs. A proteomics analysis of coculture media will help reveal the role of other important trophic factors. Different cell-cell interactions could be possible if PL-MSCs are in physical contact with HMEECs. Finally, for applications in the clinical setting, *in vivo* studies will be needed to examine changes in PL-MSCs further, after long-term transplantation to the middle ear epithelium.

## Figures and Tables

**Table 1:** Oligonucleotide primer sequences for quantitative reverse transcriptase polymerase chain reaction.

Gene	Primer sequence (forward)	Primer sequence (reverse)	Annealing temperature (°C)	Product size (bp)
MUC5B	5′-GCCTACGAGGACTTCAACGT-3′	5′-CCTTGATGACCACACGGGTG-3′	60	79
TNF*α*	5′-CCCATGTTGTAGCAAACCCT-3′	5′-TGAGGTACAGGCCCTCTGAT-3′	60	132
IL1*β*	5′-CATTGCTCAAGTGTCTGAAGC-3′	5′-CATGGCCACAACAACTGACG-3′	60	238
IL6	5′-ACTCACCTCTTCAGAACGAATTG-3′	5′-CCATCTTTGGAAGGTTCAGGTTG-3′	60	149
Cyclooxygenase 2 (COX2)	5′-CAAATTGCTGGCAGGGTTGC-3′	5′-AGGGCTTCAGCATAAAGCGT-3′	60	139
PGE2	5′-GAAGAAGGCCTTTGCCAACC-3′	5′-GACGAAGCCCAGGAAAAGGA-3′	60	145
TGF*β*	5′-GCAAGTGGACATCAACGGGT-3′	5′-TCCGTGGAGCTGAAGCAATA-3′	60	174
VEGF	5′-TCCACCATGCCAAGTGGTC-3′	5′-GTCCACCAGGGTCTCGATTG-3′	60	128

## Data Availability

The raw data of experiments used to support the findings of this study are available from the corresponding author upon request.

## Supplementary Materials

Supplementary Materials . Supplementary Figure 1: changes in TNF*α* and COX2 mRNA expression levels of placenta-derived mesenchymal stem cells (PL-MSCs) after coculture with particulate matter- (PM-) treated human middle ear epithelial cells (tumor necrosis factor alpha (TNF*α*), cyclooxygenase 2 (COX2)). Supplementary Figure 2: changes in TGF*β*, PTGES, and VEGF mRNA expression levels of human middle ear epithelial cells (HMEECs) after coculture with particulate matter- (PM-) treated HMEECs (transforming growth factor beta (TGF*β*), vascular endothelial growth factor (VEGF), and prostaglandin E synthase (PTGES)). Supplementary Figure 3: the ROS levels after 1 and 4 days after 300 of particulate matter exposures.
