# Supplementary material for: Effects of Placenta-Derived Mesenchymal Stem Cells on the Particulate Matter-Induced Damages in Human Middle Ear Epithelial Cells
Source: Stem Cells Int. 2019 Nov 14;2019:4357684. doi: 10.1155/2019/4357684 (PMC6878801; doi:10.1155/2019/4357684)

**Supplementary Materials**

**Supplementary Figure 1.** Changes in *TNFα* and *COX2* mRNA expression levels of placenta-derived mesenchymal stem cells (PL-MSCs) after co-culture with particulate matter (PM)-treated human middle ear epithelial cells. (*tumor necrosis factor alpha [TNFα], cyclooxygenase 2 [COX2]*)

**
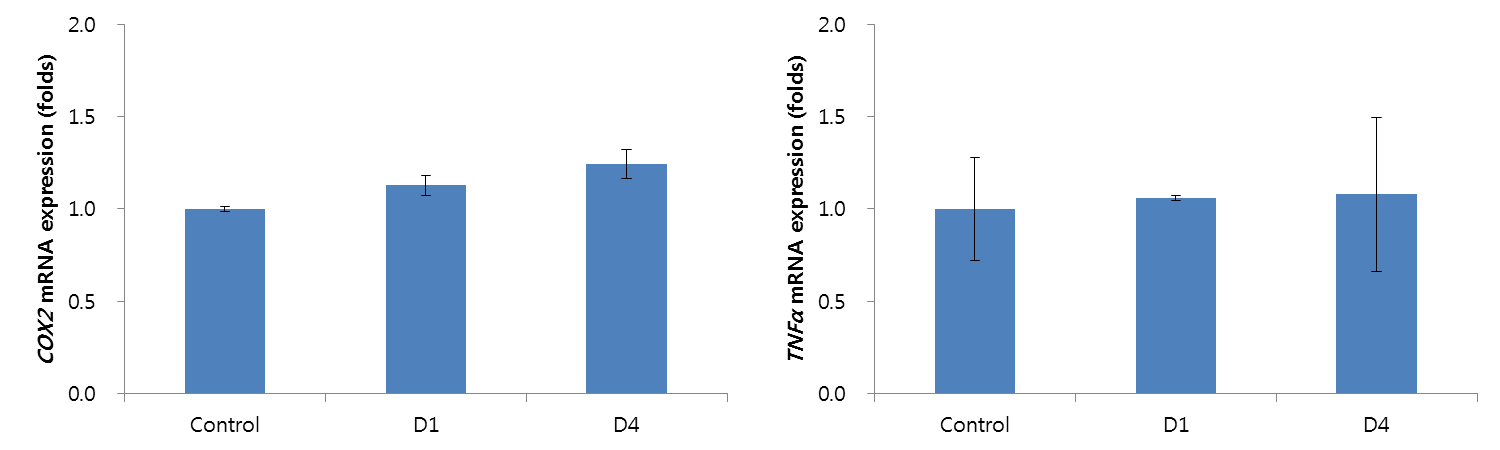
**

**Supplementary Figure 2.** Changes in *TGFβ, PTGES,* and *VEGF* mRNA expression levels of human middle ear epithelial cells (HMEECs) after co-culture with particulate matter (PM)-treated HMEECs. (*transforming growth factor beta [TGFβ], vascular endothelial growth factor [VEGF], prostaglandin E synthase [PTGES]*)


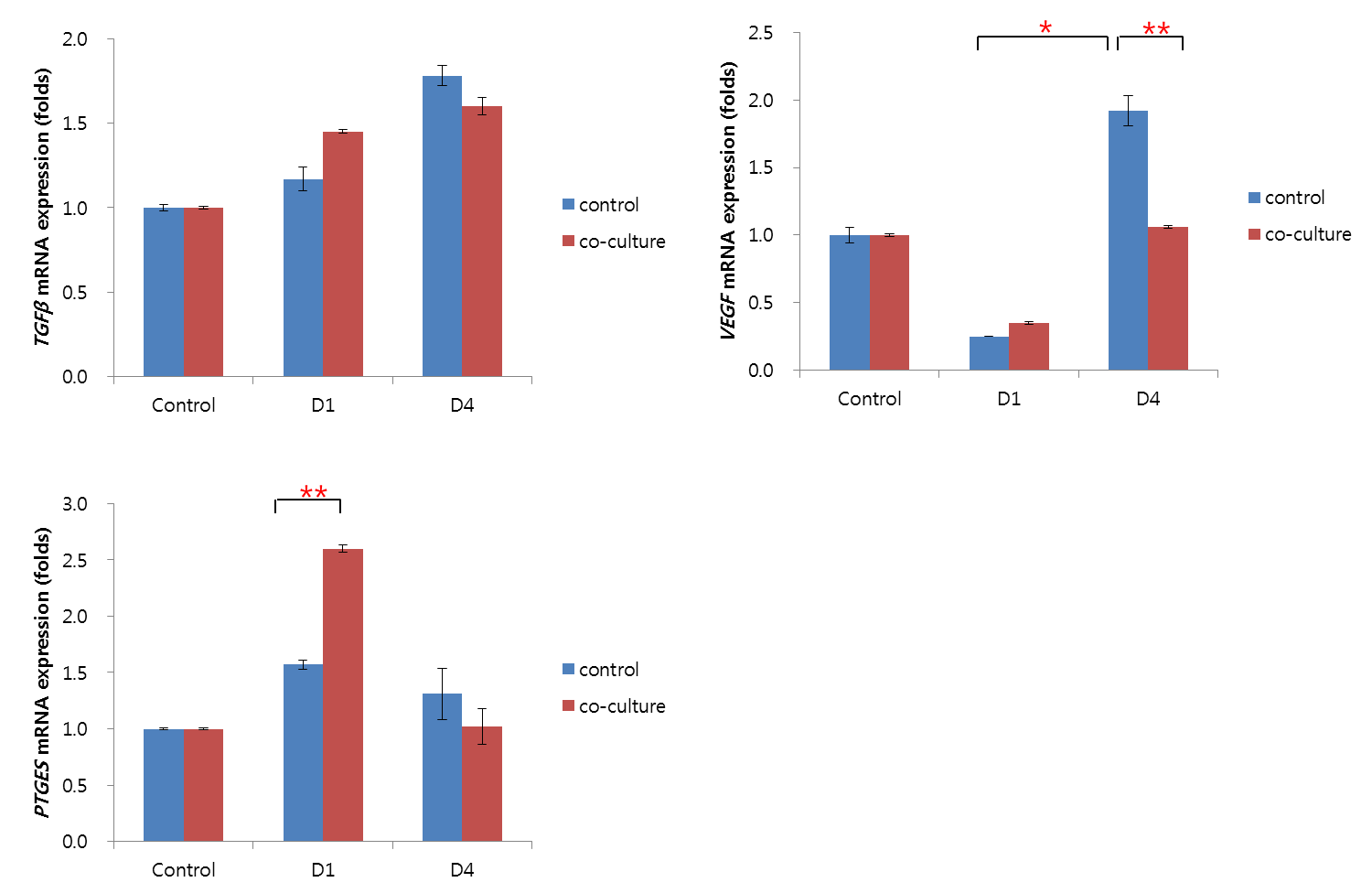


**Supplementary Figure 3.** The ROS levels after 1 and 4 days, after 300 µg/mL of particulate matter exposure.


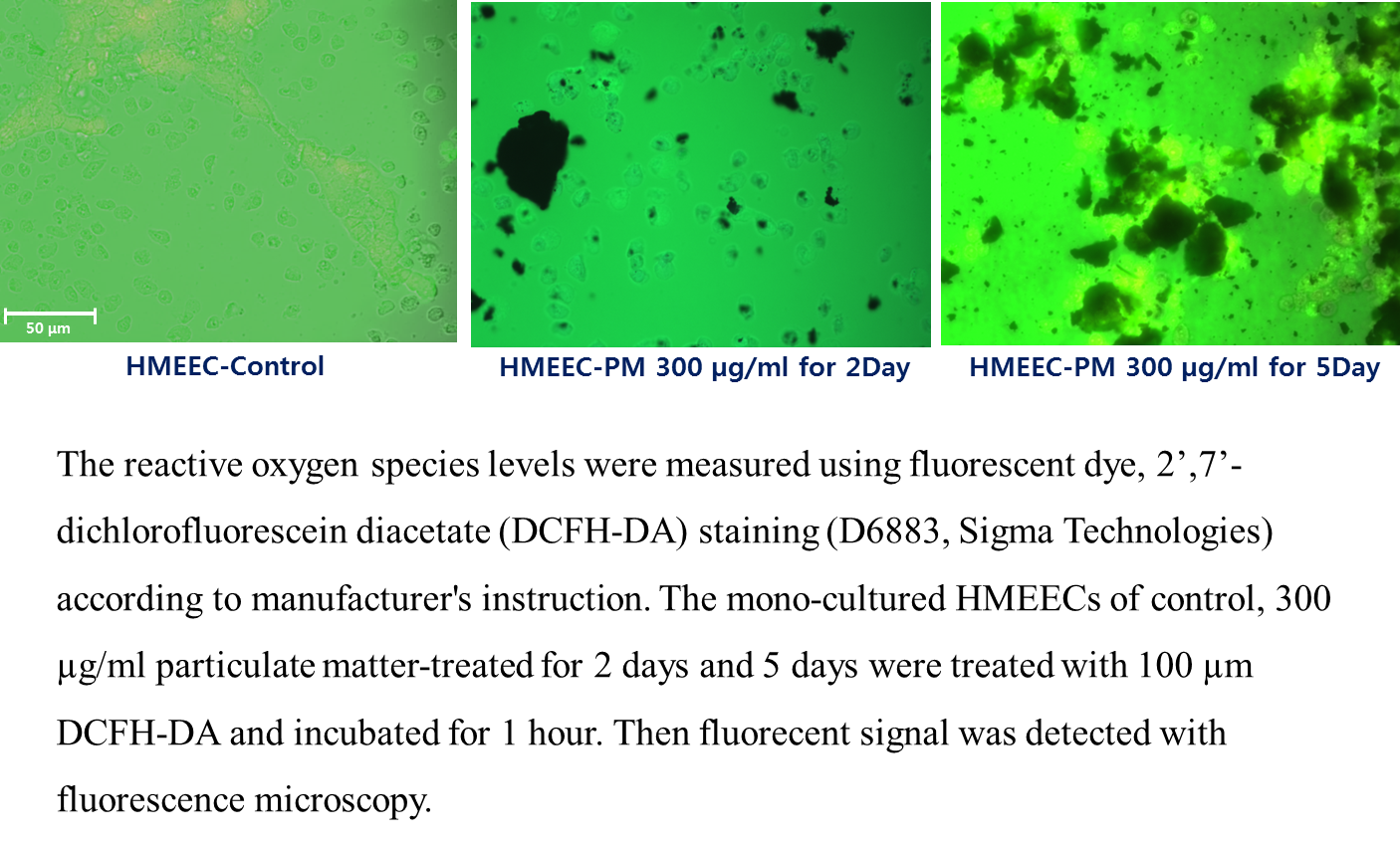

Supplement: Supplementary Materials — . Supplementary Figure 1: changes in TNFα and COX2 mRNA expression levels of placenta-derived mesenchymal stem cells (PL-MSCs) after coculture with particulate matter- (PM-) treated human middle ear epithelial cells (tumor necrosis factor alpha (TNFα), cyclooxygenase 2 (COX2)). Supplementary Figure 2: changes in TGFβ, PTGES, and VEGF mRNA expression levels of human middle ear epithelial cells (HMEECs) after coculture with particulate matter- (PM-) treated HMEECs (transforming growth factor beta (TGFβ), vascular endothelial growth factor (VEGF), and prostaglandin E synthase (PTGES)). Supplementary Figure 3: the ROS levels after 1 and 4 days after 300 of particulate matter exposures. [file 4357684.f1.docx]
